# Thermodynamic Irreversibility of the Flow and Transfer Phenomena Within Streamlined Structures of the Catalytic Reactors

**DOI:** 10.3390/e27070675

**Published:** 2025-06-25

**Authors:** Mateusz Korpyś, Adam Rotkegel, Anna Gancarczyk, Marzena Iwaniszyn, Katarzyna Sindera, Mikołaj Suwak, Andrzej Kołodziej

**Affiliations:** 1 Institute of Chemical Engineering, Polish Academy of Sciences, Bałtycka 5, 44-100 Gliwice, Poland; arot@iich.gliwice.pl (A.R.); anna.g@iich.gliwice.pl (A.G.); miwaniszyn@iich.gliwice.pl (M.I.); katarzyna.sindera@iich.gliwice.pl (K.S.); mikolaj.suwak@iich.gliwice.pl (M.S.); ask@iich.gliwice.pl (A.K.); 2 Faculty of Civil Engineering and Architecture, Opole University of Technology, Katowicka 48, 45-061 Opole, Poland

**Keywords:** short-channel structures, heat transfer, flow friction, computational fluid dynamics, entropy production, irreversible thermodynamics

## Abstract

An analysis is presented of the irreversibility of flow and thermal phenomena in innovative streamlined structured packing of catalytic chemical reactors. The fundamental equations of irreversible thermodynamics defining entropy production as a result of flow friction and heat transport are formulated. The parameters describing the flow and heat transport in these equations are determined using the Computational Fluid Dynamics (CFD) methodology. Local entropy production due to flow friction and heat transport in the channel structure is plotted and compared with flow-temperature maps and relations for flow resistance, pressure gradient, and Nusselt number derived from CFD. The calculations were performed for three gas velocities: 0.3; 2.0, and 6.0 ms^−1^. It was found that the entropy due to flow friction increases strongly with increasing gas velocity, while the entropy due to heat transport decreases with gas velocity, but to a limited extent. These opposing tendencies mean that there is always a minimum of the total entropy production (including these due to flow friction and heat transport), usually for moderate gas velocity. This minimum constitutes the optimum operating point of the reactor from the thermodynamic point of view.

## 1. Introduction

Recently, catalytic structured reactors of divers structured internals have become standard in industrial and environmental applications. Historically, the first approach was monolithic, made of ceramic (cordierite) and consisting of many straight, parallel capillary channels. Soon, other materials like graphite, alumina, and metals appeared [1], as well as other structures, e.g., wire gauzes and solid foams. The basic requirements for structured catalytic reactors are low fluid flow resistance, large specific surface area, and intensive heat and mass transport to the catalyst deposited on the surface of the structure. In capillary channels, the fluid flow is usually laminar; this implies low flow resistance. The significant specific surface area of the structured packing, which translates into the amount of catalyst involved in the reaction, is basically limited mainly by the structure production technology. However, the heat and mass transfer in laminar flow take place via conduction and diffusion, respectively, and thus their intensity is often unsatisfactory.

In long capillary channels, fully developed laminar flow occurs, characterized by parallel fluid streamlines or velocity vectors. The heat (mass) transport intensity, measured by Nusselt (Sherwood) numbers, is high only near the channel inlet, but further down the channel, it decreases asymptotically to a constant value that depends on the shape of the channel cross-section and the boundary conditions on its wall [2]. This asymptotic value does not depend on the fluid velocity (Reynolds number). Note that although higher flow resistance can be overcome at the expense of higher pumping energy, it is not possible to simply increase the intensity of mass and heat transport. The mass transport is essential here: only those molecules that reach the catalyst surface can react, and the heat of reaction must be transferred between the surface and the fluid. Note that the paradigm of the heat and mass transfer analogy (Chilton–Colburn equation) bounds strictly the heat and mass transfer intensity. In this situation, attempts are made to change the structure in a way that ensures higher transport intensity. One possibility is the so-called short-channel structures presented in previous works [3]; the idea is to shorten the channels so that the area of developing laminar flow covers almost the entire (very short) capillary channel of the structure.

The interest in structured catalysts is still growing. The goal is to obtain a large surface area for the catalyst layering, coupled with intense heat and mass transfer characteristics. In addition to the monolithic reactors that are still being studied, solid, metal, and ceramic foams have appeared, used as catalyst carriers. Monoliths with short channels are being studied; in the work Gribovskiy et al. [4], a catalytic multichannel reactor with short channels (10 mm in length and 2 mm diameter) is investigated for methanol steam reforming. Wang et al. [5] presented a novel-structured short-channel Pt-catalyst deposited on an aluminum support. Wang et al. [6] used a combination of twist and helix in helical-twisted tubes to enhance heat transfer

Recently, a short-channel streamlined structure has been developed, the walls of which are shaped like an airplane wing (airfoil profile) [7,8].

Mathematical modelling of heat transport issues in laminar flow was started by Graetz [9,10]; a number of solutions for increasingly advanced problems are presented in the book of Shah and London [2]. In this classic modelling, flat velocity and temperature profiles are assumed at the channel inlet; down the channel, they transform into parabolic profiles, typical of developed laminar flow. However, this approach does not take into account the thickness of the channel walls, which causes the streamlines to deviate from the axis and, as a result, to create flow patterns such as the inlet vortex, the stream split point, and the outlet vortex [7,8]. The visualization and study of the flow patterns mentioned above became possible thanks to the use of CFD (Computational Fluid Dynamics). In particular, the inlet vortex causes a significant reduction in transport intensity in the inlet region, where, according to the theory, transport should be the most intense [2]. The streamlined structures mentioned above have been designed to avoid the inlet vortex. The velocity vectors are tangent to the airfoil-like (rounded) leading edge of the channel wall and the inlet vortex is not formed [7,8].

Catalytic structured reactors are used in many technology fields, including the chemical and energy industries. They are of particular importance in the area of environmental protection. As mentioned above, the first area of application of monoliths was the afterburning of car exhaust gases, and structured reactors are still used for the afterburning of various substances emitted into the atmosphere. Low concentrations of the combusted substances characterize the vast majority of such emissions. This causes problems with thermal operations within the whole apparatus, including maintaining the autothermal process and recovering excess heat.

More detailed discussion on the problems of laminar flow and transfer phenomena is presented in reference [8]. The streamlined structure, square in cross-section and 3 mm long, was studied experimentally as well as using the CFD simulation of the flow and transfer behavior. The experiments reflected the macroscopic functioning of the structure (including average heat transfer and flow resistance) and allowed verification of the CFD simulation’s accuracy. The CFD enabled deeper insight into the flow and transfer phenomena. The flow patterns were identified within a wide range of Reynolds number. Longitudinal and transverse distributions of heat transport intensity (Nusselt numbers) were determined in reference [8].

However, in our opinion, the innovative structure requires deeper analysis of the process phenomena in the light of the process irreversibility. The two-law thermodynamic analysis of the chemical reactors and other apparatus realizing the heat/mass transfer and fluid flow operations, covering mainly entropy production as a function of process parameters, is presented in many works. Kong et al. [11] analyzed a membrane reactor designed for hydrogen iodide decomposition and modelled entropy generation due to chemical reaction, heat, and mass transfer. The numerical study of Wang et al. [6] presents the entropy production as a result of irreversible heat and mass transport and a chemical reaction. Abidi-saad et al. [12] numerically analyzed thermodynamic characteristics of convection heat transfer and fluid flow in vertical convergent channel. Kizilova et al. [13] consider the minimum entropy production concept for optimization of tubular chemical reactors with a cooling system. Similar works devoted to the analysis of entropy production and irreversibility of phenomena are currently presented, e.g., references [14,15,16].

Irreversible thermodynamics enables the analysis of phenomena in terms of their distance from the equilibrium state. In reference [17], such an analysis was presented for catalytic reactors of various types. Entropy production was taken into account here in all basically irreversible phenomena occurring in structured catalytic reactors: chemical reaction (in this case the reaction of organic compounds combustion), diffusion of reactants, heat transport, and flow friction (viscous dissipation of energy). Entropy production is here a measure of the distance from the equilibrium state, i.e., in a sense, thermodynamic imperfection. All the mentioned phenomena occur at a certain distance from the equilibrium state, with a finite driving force, and the entropy produced in them depends in different ways on, for example, the process temperature or flow rate. The sum of entropy production as a result of all these phenomena indicates a distance from the equilibrium; the minimum of the summarized entropy indicates the optimal operating point.

This paper presents analysis of the entropy production due to the viscous flow friction and heat transfer in the streamlined structures [8]. The purpose of this analysis is not only to determine the value of entropy produced as a result of heat transport and flow friction, and it is not the main goal to determine for a specific structure the optimal operating point, that is, the minimum of the entropy produced. The CFD results presented in reference [8], including distributions of gas pressure, heat transfer coefficients, and temperature-flow maps, allow for determining the locally produced entropy and linking it with transport and flow phenomena. In particular, it is possible to observe how the generated vortices and heat transport minima and maxima affect the local entropy production and thus its total production, i.e., the efficiency of the considered structure.

## 2. Basic Equations of Irreversible Thermodynamics of Processes

The process under consideration is here fluid flow inside a capillary channel of the considered streamlined structured catalyst carrier, square in cross section, accompanied by heat transfer between channel wall and the flowing fluid [8]. Both the processes are obviously irreversible, and thus entropy of the considered system increases due to viscous dissipation of energy (viscous friction) and heat transfer. Such an entropy increase is called entropy production [18,19]. Entropy is not produced at equilibrium (or during a quasi-static process running infinitely close to the equilibrium).

The thermodynamic calculations presented in the paper are based on our previous study on the streamlined structure [8] where the flow patterns were illustrated and discussed using CFD. There, the flow resistance (Fanning friction factor) and heat transfer (Nusselt number) were studied using both CFD and experiments. The results obtained in reference [8], like heat transfer coefficients and friction factors, were used to perform thermodynamic calculations in accordance with the methodology presented below.

During modelling of the structure, which is intended to be a catalyst carrier in a chemical reactor, we consider a single channel, in which viscous laminar flow and heat transport from the channel wall to the flowing fluid take place with the boundary condition H1 according to reference [2], i.e., constant heat flux q. The entropy produced in the entire channel space is denoted as S, W∙K^−1^; the entropy generated locally per unit length of the channel is S′, W∙m^−1^ K^−1^; per unit area of the channel wall is S″, W∙m^−2^ K^−1^; per unit volume (locally) is S‴, W∙m^−3^ K^−1^. Note that S‴A=S″P=S′ assuming entropy production is constant within a considered space.

In the literature of irreversible thermodynamics, entropy production is calculated as the product of a stream $J_{n}$ and a driving force $\Delta \pi_{n}$ (causing the stream) divided by the process temperature T [19]:

(1) $$S_{n}=\frac{J_{n}{\Delta \pi }_{n}}{T}.$$

Assuming the stream $J_{n}$ is proportional to the driving force:

(2) $$J_{n}=k_{n}{\Delta \pi }_{n},$$

The entropy production is proportional to the square of the driving force $\;\Delta \pi_{n}$. The subscript n refers to the process producing entropy. The entropy production is considered due to the following irreversible phenomena:

- Flow friction, i.e., work performed against the flow resistance—subscript F; the stream here is the mass flow rate, $\dot{m}$, and the driving force is pressure gradient causing the stream;
- Heat transfer between flowing fluid and channel wall (structure)—subscript H; the stream here is the heat flux, q, and the driving force is a temperature gradient.

Total entropy production is the sum of both the components considered:

(3) $$S_{P}=S_{H}+S_{F}.$$

The entropy per unit channel length produced due to viscous dissipation of energy, $S_{F}^{\prime }$, can be expressed as the work against flow resistance, thermally dissipated to the surroundings:

(4) $$S_{F}^{\prime }=\frac{1}{T}\left(-\frac{dp}{dx}\right)Aw=\frac{2fA}{T}\frac{w^{3}\rho }{D_{h}}.$$

Note that the equation is identical, with at most formal differences, to the equations presented in the papers [17,20]. A denotes the cross-sectional surface area perpendicular to the channel axis (and thus to the general flow direction).

**Figure 1:**
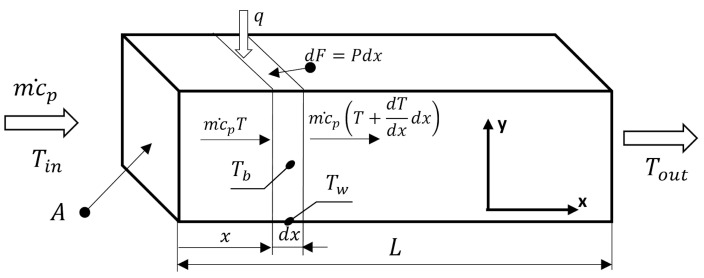
Scheme of the thermal balance of the channel.

The scheme of the thermal balance of the channel is presented in Figure 1. The entropy produced per unit volume elsewhere in the channel due to heat transfer is as follows:

(5) $$S_{H}^{\prime \prime \prime }=\frac{-q}{T^{2}}grad\;T=\frac{-q}{T^{2}}\frac{dT}{dy}=\frac{\lambda }{T^{2}}{\left(\frac{dT}{dy}\right)}^{2},$$

assuming that whole heat transport takes place perpendicularly to the channel wall, i.e., along the y axis. According to Equation (2), $q=-\lambda \left(dT/dy\right)$, we obtain the following:

(6) $$-\frac{dT}{dy}=\frac{q}{\lambda },$$

where λ is the fluid thermal conductivity, and the local entropy production per unit channel volume $S_{H}^{\prime \prime \prime }$ is as follows:

(7) $$S_{H}^{\prime \prime \prime }=\frac{\lambda }{T^{2}}{\left(\frac{dT}{dy}\right)}^{2}=\frac{q^{2}}{{\lambda T}^{2}}.$$

Entropy related to 1 m^2^ of the channel wall area, $S_{H}^{\prime \prime }$, can be calculated by integrating Equation (7) along the y axis transverse to the direction of flow. Herwig [20] used the concept of laminar thermal boundary layer with a certain equivalent thickness δ, in which linear fluid temperature distribution is assumed from bulk temperature $T_{b}$ to wall temperature $T_{w}$.

(8) $$S_{H}^{\prime \prime }=\int_{0}^{\delta }S_{H}^{\prime \prime \prime }dy=\int_{0}^{\delta }\frac{q^{2}}{{\lambda \left[T(y)\right]}^{2}}dy,$$

where the following applies:

(9) $$T=T_{w}-\frac{T_{w}-T_{b}}{\delta }y.$$

The slope coefficient of the above equation is the temperature gradient at the channel wall, thus giving the following:

(10) $$q=\frac{\lambda }{\delta }\left(T_{w}-T_{b}\right)=\alpha \left(T_{w}-T_{b}\right).$$

Integration of Equation (8) with substitution of Equations (9) and (10) gives the following:

(11) $$S_{H}^{\prime \prime }=q\frac{T_{w}-T_{b}}{T_{w}T_{b}}.$$

The equation, close to that presented in [20], was used for numerical integration along the channel to derive the whole entropy produced due to the heat transfer:

(12) $$S_{H}=\int_{0}^{F}S_{H}^{\prime \prime }dF=\int_{0}^{L}{PS}_{H}^{\prime \prime }dx,$$

where P is the channel perimeter, L is length, and F is the surface area of the channel wall; the results are presented in the next sections.

The thermal balance between the heat transferred between the channel wall and the bulk stream is, according to Figure 1, as follows:

(13) $$\dot{m}c_{p}\frac{dT_{b}}{dx}=qP.$$

Assuming negligible heat losses and axial conduction. This, with Equation (10) introduced, leads to the formula for local bulk temperature $T_{b}$.

(14) $$T_{b}=T_{in}+\frac{qP}{\dot{m}c_{p}}\;x,$$

A simple relation between the bulk and wall temperatures $T_{b}$. and $T_{w}$ is as follows:

(15) $$T_{w}=\frac{q}{\alpha }+T_{b}.$$

Substituting Equation (15) into Equation (11) gives the following:

(16) $$S_{H}^{\prime \prime }=\frac{q^{2}}{\alpha T_{b}}\frac{1}{T_{b}+\frac{q}{\alpha }}.$$

Bulk temperature can be calculated from the balance (14) or, more precisely, from the CFD data as the mix-cup temperature. Heat flux q is constant, and local heat transfer coefficient α can be taken from the Nusselt number Nu distribution derived using CFD:

(17) $$Nu=\frac{\alpha D_{h}}{\lambda }.$$

Note that the channels within the streamlined structure have non-equal dimensions; th us, equivalent diameter $D_{h}$, cross-sectional area A, and local velocity w differ along the channel.

Another approach to derive entropy $S_{H}$ produced in the channel is substituting Equation (6) into Equation (5) thus giving the local entropy production per unit channel length:

(18) $$S_{H}^{\prime }=\frac{dS_{H}}{dx}=\frac{q^{2}A}{{\lambda T}^{2}}.$$

Integrating Equation (18) along the reactor gives the total entropy produced in reactor due to heat transfer $S_{H}$:

(19) $$S_{H}=\frac{q\dot{m}c_{p}}{\lambda a}\left(\frac{1}{T_{in}}-\frac{1}{T_{out}}\right)=\frac{q\dot{m}c_{p}}{\lambda a}\frac{T_{out}-T_{in}}{T_{out}T_{in}},$$

where a is specific surface of the channel:

(20) $$a=\frac{F}{V}=\frac{PL}{AL}=\frac{P}{A}.$$

The total entropy produced in the channel (neglecting channel geometry and the flow nature) is given by Equation (3) with Equations (4) and (12) or Equation (19) introduced.

Note that the entropies produced by flow friction, $S_{F}$, and heat transfer, $S_{H}$, show an opposite dependence on the flow rate. $S_{F}$ increases with flow rate, which is directly visible in Equation (4). $S_{H}$, however, decreases with the flow rate due to the increasing heat transfer coefficient that strongly influences heat flux q and the temperature field in the channel, and thus the temperature gradient as well. This opposite behavior leads to a certain minimum of the overall entropy  S from Equation (3); the minimum can be assumed as the optimum for the considered case (including channel geometry, flow rate, fluid parameters, temperature distribution, heat flux, etc.). Below, the results of the entropy calculations are presented and discussed.

## 3. Reactor Structured Internals Studied

**Figure 2:**
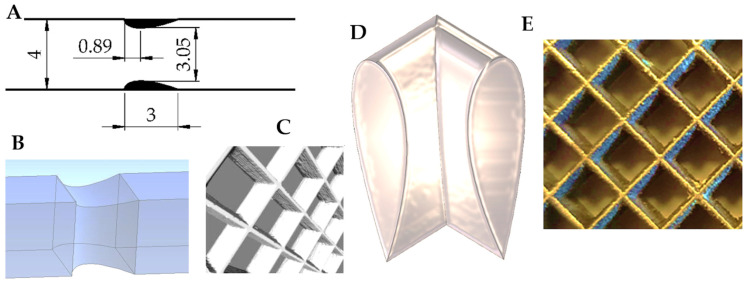
Pictures of square streamlined internals studied. (**A**)—Essential dimensions; (**B**)—channel—computational domain for CFD simulations; (**C**)—computer tomography of the SLM manufactured structure (reconstruction); (**D**)—CAD design, cutting out the channel corner; (**E**)—photo of the manufactured structure.

The square streamlined structure studied is presented in Figure 2. Figure 2C shows a fragment of the structure consisting of several square channels connected into a larger matrix (computed tomography). This structure was manufactured using the SLM (Selective Laser Melting) method from stainless steel, then experimentally studied [8].

Figure 2A presents essential dimensions of the structure. Note that the physically existing structure forming the channel is represented by two short airfoil-like black shapes, i.e., cross-sections of the 3 mm long channel walls.

The geometrical data of the structure (the same as of the single channel) are as follows: specific surface area a=925 m^−1^; porosity (void fraction) ε=0.71; hydraulic diameter $D_{h}=4\varepsilon /a=0.0031$ m; square channel length 3 mm. More information about the streamlined structures and their properties is available in [8].

## 4. Methods of Investigations

Airflow and heat flow simulations in the channel were performed using the CFD methodology, applying the Ansys Fluent software. A numerical grid consisting of 24.6 million polyhedral elements was created. Calculations were performed in steady state, with defined constant values (independent of temperature) of physical parameters: density, 1.225 kg∙m^−3^; viscosity coefficient, 1.7894∙10^−5^ Pa∙s; thermal conductivity coefficient, 0.0242 W m^−1^ K^−1^; specific heat, 1006.43 J kg^−1^ K^−1^.

The second-order discretization was used in the calculations for all equations. The Least-Squares Cell-Based method and SIMPLE algorithm (Semi-Implicit Method for Pressure-Linked Equations) were used to calculate the gradients.

During the simulations, a constant velocity boundary condition at the inlet and a pressure-outlet condition were used. The flow was perpendicular to the inlet cross-section, and the velocity profile was uniform. Flow velocities of 0.3, 2, and 6 m s^−1^ were analyzed. A symmetry condition was applied to the walls of the computational domain, except for structures. A constant energy flux of 1500 Wm^−2^ was implemented on the channel walls. At the time of completion of the calculations, the residuals for all equations were below 10^−4^, and for the energy equation below, 10^−7^. The accuracy of the CFD methodology with respect to streamlined structures has been experimentally confirmed, e.g., in reference [8].

To calculate entropy production according to the equations given in Section 2, the results of CFD simulations of flow-thermal phenomena in the tested channel were used. The 3 mm long channel was divided into 30 slices of 0.1 mm thickness, which resulted in 31 parallel planes perpendicular to the flow direction, spaced every 0.1 mm along the length of the channel (x axis). These planes intersect mesh cells, and at the intersection points, values calculated in Fluent were generated, such as: pressure, temperature, velocity. There were from about 20,000 to 40,000 such points on each plane. Due to the uneven distribution of mesh cells, as well as the different number of data points on each plane, each plane was normalized, dividing it into 100 × 100 identical unit surfaces. The data points on each of them were averaged and the resulting value was considered the average for a given unit area.

For further numerical calculations, the Matlab R2019 program was used. The entropy production due to flow friction $S_{F}$ was calculated by integrating the derivative relation $S_{F}^{\prime }$, Equation (4), over the channel length using the trapezoidal method. The term $\frac{1}{T}\left(-\frac{dp}{dx}\right)Aw$ in Equation (4) was calculated as the integral:

(21) $$\iint_{0}^{A}\frac{1}{T}\left(-\frac{dp}{dx}\right)wdA,$$

which can be approximated as the product of the area A and the average value $\frac{1}{T}\left(-\frac{dp}{dx}\right)w$ of all unit areas (10,000 in number) in the plane:

(22) $$\frac{1}{T}\left(-\frac{dp}{dx}\right)wA=\frac{A}{10,000}\sum_{i=1}^{100}\sum_{j=1}^{100}\left[\frac{1}{T_{i,j}}{\left(-\frac{dp}{dx}\right)}_{i,j}w_{i,j}\right],$$

where

(23) $${⋀}_{i,j\;\in <1,100>}{\left(\frac{dp}{dx}\right)}_{i,j}=\frac{p_{i,j,k+1}-p_{i,j,k}}{x_{k+1}-x_{k}}.$$

The entropy production due to heat transfer $S_{H}^{\prime }$ was calculated by integrating Equation (16) using the trapezoidal method, taking into account that the fluid bulk temperature $T_{b}$ is given by Equation (14).

## 5. Results and Discussion

CFD studies were performed for a single channel of the streamlined structure 3 mm long. In the CFD simulations, the boundary condition of constant heat flux on the filling surface was assumed, i.e., condition H1 [2], and three superficial gas velocities were assumed for calculations, i.e.,: $w_{0}$ = 0.3 m∙s^−1^; 2 m∙s^−1^; 6 m∙s^−1^, respectively. The gas parameters were assumed as for air at atmospheric pressure and at local temperature for the considered point of the structure channel.

**Figure 3:**
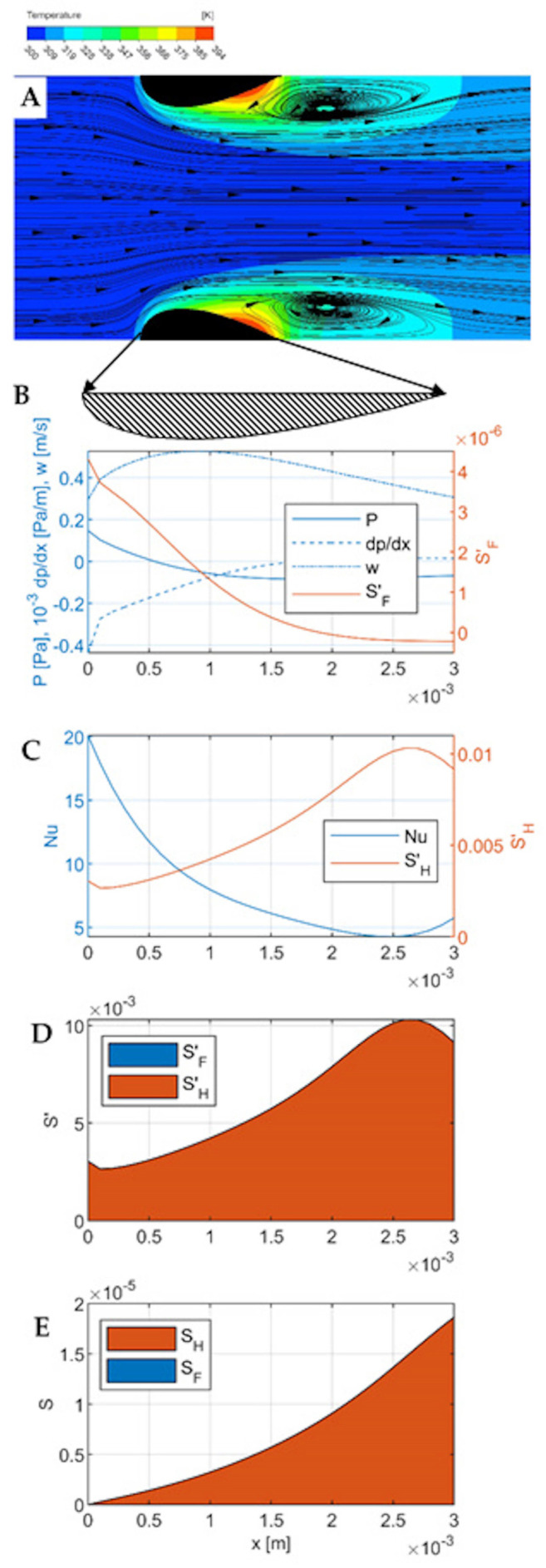
Results for gas velocity $w_{0}=$ 0.3 m∙s^−1^. (**A**)—Flow-temperature map; (**B**)—distributions of gas velocity, pressure, pressure gradient, and local entropy production due to flow friction $S_{F}^{\prime }$ along the channel; (**C**)—distribution of Nusselt number and local entropy production due to heat transfer $S_{H}^{\prime }$ along the channel; (**D**)—comparison of local entropy production $S_{F}^{\prime }$ and $S_{H}^{\prime }$; (**E**)—comparison of cumulative entropy production due to flow friction and heat transfer (integrated Figure (**D**)).

**Figure 4:**
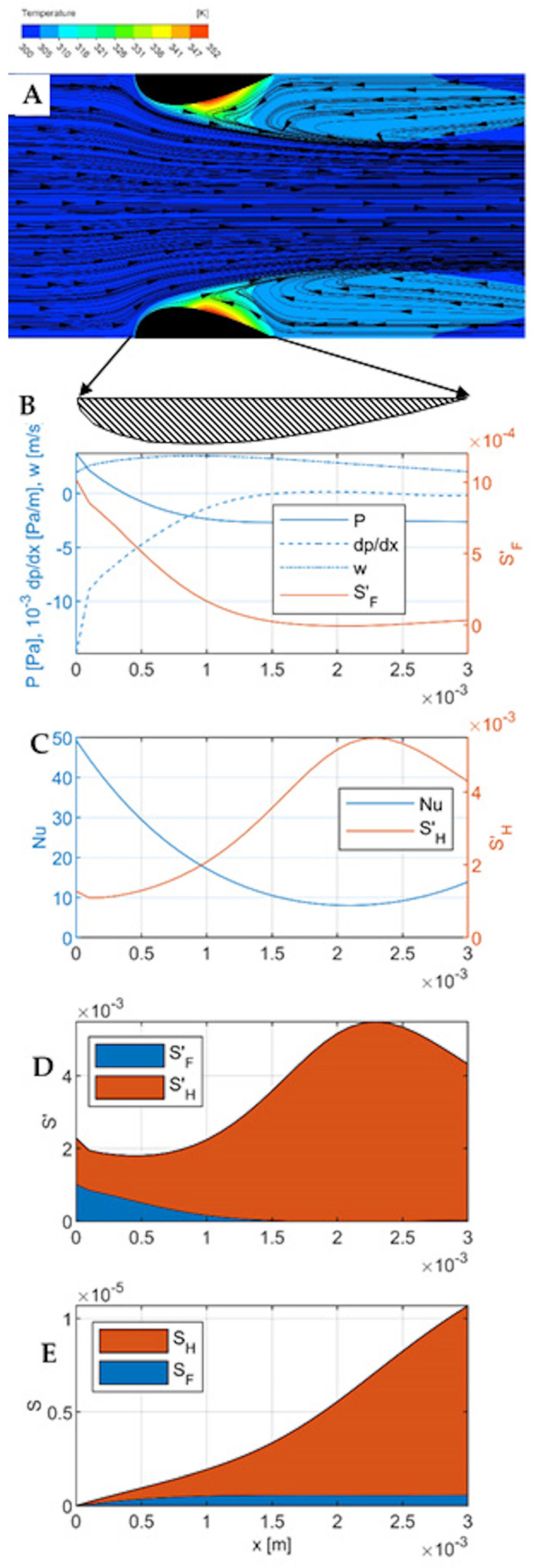
Results for gas velocity $w_{0}=$ 2 m∙s^−1^. (**A**)—Flow-temperature map; (**B**)—distributions of gas velocity, pressure, pressure gradient, and local entropy production due to flow friction $S_{F}^{\prime }$ along the channel; (**C**)—distribution of Nusselt number and local entropy production due to heat transfer $S_{H}^{\prime }$ along the channel; (**D**)—comparison of local entropy production $S_{F}^{\prime }$ and $S_{H}^{\prime }$; (**E**)—comparison of cumulative entropy production due to flow friction and heat transfer (integrated Figure (**D**)).

**Figure 5:**
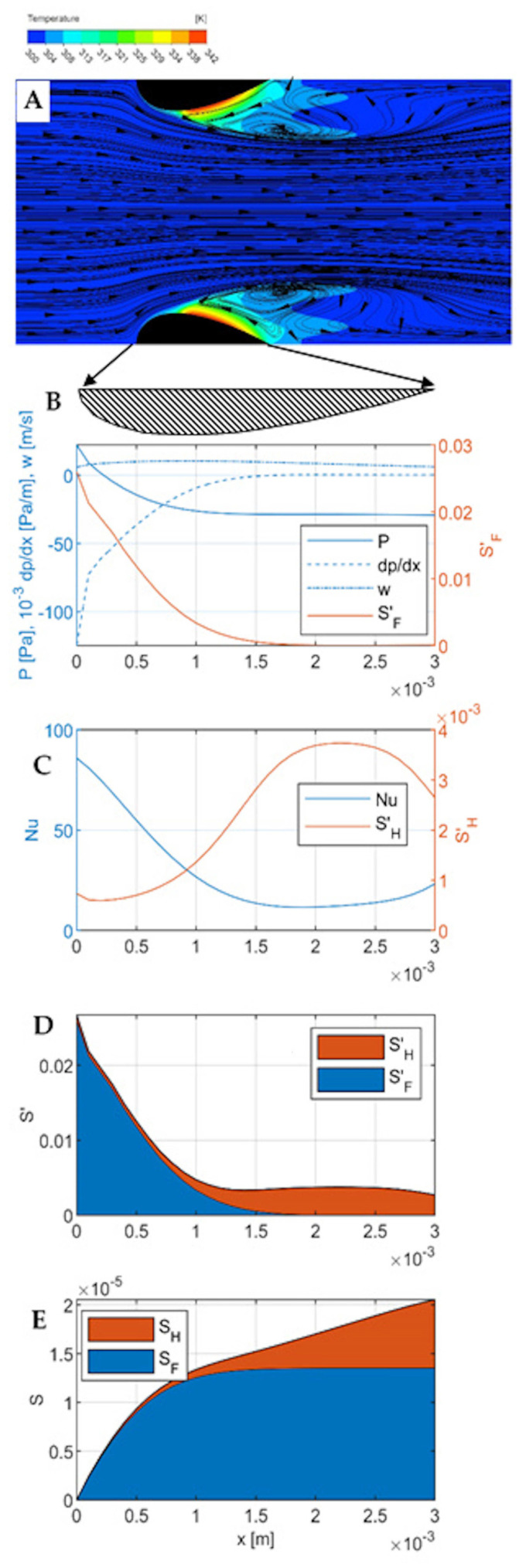
Results for gas velocity $w_{0}=$ 6 m∙s^−1^. (**A**)—Flow-temperature map; (**B**)—distributions of gas velocity, pressure, pressure gradient, and local entropy production due to flow friction $S_{F}^{\prime }$ along the channel; (**C**)—distribution of Nusselt number and local entropy production due to heat transfer $S_{H}^{\prime }$ along the channel; (**D**)—comparison of local entropy production $S_{F}^{\prime }$ and $S_{H}^{\prime }$; (**E**)—comparison of cumulative entropy production due to flow friction and heat transfer (integrated Figure (**D**)).

The results of CFD simulations and the modelling of entropy production are presented for the velocities of 0.3 m∙s^−1^, 2 m∙s^−1^, and 6 m∙s^−1^ in Figure 3, Figure 4 and Figure 5, respectively. Each Figure contains five parts numbered from A to E.

Part A is a temperature-flow map of the channel, determined using CFD and showing the fluid flow lines and temperatures within the channel, as well as some distance before the inlet and beyond the outlet. All the quantities used in the calculations (including pressure, local velocity, temperature) were determined in CFD simulations. All the flow-temperature maps in Figure 3, Figure 4A and Figure 5A are projected onto a plane that is the diagonal of the channel.

Part B shows the distribution of the essential flow parameters along the channel: local velocity, pressure, pressure gradient, and local entropy produced due to flow friction per unit length of the channel $S_{F}^{\prime }$. All these quantities are calculated for successive cross-sections of the channel (taking into account the variable dimensions of these cross-sections), where each cross-section is a matrix of 100 × 100 elements, and successive cross-sections are spaced 0.1 mm apart (which gives 31 cross-sections within the channel). The quantities given in the graphs are averages for a given cross-section. These calculations do not include the area before and beyond the channel because heat transport occurs exclusively within the channel.

Part C presents thermal quantities—the distribution along the channel of the local Nusselt number and the local entropy produced due to heat transfer per unit length of the channel $S_{H}^{\prime }$.

Part D compares the local entropy produced due to flow friction, $S_{F}^{\prime }$, and due to heat transfer, $S_{H}^{\prime }$ in one graph. Part E contains the integrals of the graphs on D and shows the cumulative entropy production $S_{F}$ and $S_{F}$ along the channel, giving a comparison of their contributions to the total entropy production in the channel region.

All graphs B–E are relative to the length of the channel, shown in Figure A as a blackened cross-section of the streamlined walls. Graphs B-D are wider than the channel, as indicated by the arrows leading to the enlarged cross-section of the wall under Figure A.

Flow-temperature map, velocity $w_{0}=$ 0.3 m∙s^−1^ (Figure 3A). For this small velocity, the flow is laminar. However, this does not exclude the occurrence of phenomena caused by inertia forces. Examples are large vortices with parallel, smooth streamlines that form behind the outlet of the channel (and also in its final part). A kind of bow wave is formed on the frontal surfaces of the streamlined. The object (the channel wall) is small (millimeter sized) and the gas velocity is moderate (up to 6 m∙s^−1^); thus, the flow is to some extent laminar, and thus phenomena such as flow separation or Karman vortex street rather do not occur [21]. The streamlined shape of the walls minimizes the bow wave, maintaining intensive heat transfer in its area. There is also no inlet vortex. However, outlet vortices are formed which have a wake character to some extent. In the central part of the channel (near the axis), the flow is laminar. In the middle of the channel length, the main (central) fluid stream meets the reversing outlet vortex. In the inlet part of the channel, especially on the frontal surfaces, intensive heat transport (significant temperature gradient) is visible, a result of intensive flow shear. Down the channel, this gradient decreases, and in the outlet vortex (final part of the channel, approx. 1/3 of the length), a hot spot is visible, indicating poor heat transfer.

Pressure and flow (Figure 3B). The mean flow velocity reaches a maximum of 0.5 ms^−1^ at about 1/3 of the channel length, and further down the channel velocity decreases to less than 0.3 ms^−1^. In the middle of the channel length, the static pressure reaches a minimum then stabilizes. The pressure gradient (negative) is the highest at the channel inlet, then decreases and stabilizes. At the channel inlet, there is a visible kink in the pressure, fluid velocity, and pressure gradient distribution lines, corresponding to the edge of the bow wave. Local entropy distribution due to flow friction is the highest at the channel inlet; down the channel, it decreases significantly. A kink near to the bow wave edge is distinct at the $S_{F}^{\prime }$ line.

Heat transport (Figure 3C). The Nusselt number reaches a maximum at the inlet to the channel, then decreases, reaching a minimum at a distance of 2.5 mm from the inlet (i.e., at 5/6 of the channel length); in the final section, it increases slightly due to the effect of the outlet vortex on the channel surface near the outlet. Entropy production due to irreversible heat transport is inversely proportional to the heat transport intensity (i.e., Nusselt number); entropy maxima correspond exactly to Nusselt number minima and vice versa. At the point corresponding to the edge of the bow wave, a small local minimum is visible on the entropy production line $S_{H}^{\prime }$, in the form of a sharp break in the line. It has no counterpart in the Nusselt number distribution.

Comparison of local entropy production due to friction and heat transfer (Figure 3D) shows that for small velocity of 0.3 m∙s^−1^, entropy due to transfer clearly dominates. The same conclusion is given from a comparison of Figure 3B,C—entropy due to friction is three orders of magnitude lower than that due to heat transfer. The cumulative entropy production is shown in Figure 3E (integrated distributions from Figure 3D). Here, too, practically all entropy comes from irreversible heat transport; total entropy produced due to heat transfer $S_{H}≅$ 1.8∙10^−5^ W K^−1^. Such small flows are clearly unfavorable for heat transport, which proceeds far from the equilibrium state.

Flow-temperature map, velocity $w_{0}=$ 2 m∙s^−1^ (Figure 4A). For this velocity, the inertial effects are more pronounced. The outlet vortices are larger, more extended outside the channel in the flow direction. In the central part of the channel (near the axis) the flow is faster, a kind of jet is created, narrowing in the flow direction, limited by larger outlet vortices. The meeting point of the central fluid stream with the outlet vortex is shifted towards the channel inlet. Heat transport at the channel inlet, especially on the frontal surfaces, is more intensive (larger temperature gradient). Down the channel, this gradient decreases. In the outlet part of the channel, there is a hot-spot, shifted towards the channel inlet in relation to the lower velocity ($w_{0}=$ 0.3 m∙s^−1^).

Pressure and flow (Figure 4B). The mean flow velocity reaches a maximum near the point where the channel cross-section is the smallest, but the velocity profile is flatter than for the velocity of 0.3 m∙s^−1^. A little further down the channel, the static pressure reaches a minimum and stabilizes. The pressure gradient is the highest at the channel inlet, then decreases and stabilizes. At the channel inlet, there is a visible kink in the pressure, fluid velocity, and pressure gradient distribution lines, similar to Figure 3B, corresponding to the edge of the bow wave. The distribution of entropy production due to the flow friction (Equation (4)) is analogous to Figure 3B, but its values are more than two orders of magnitude larger.

Heat transport (Figure 4C). The Nusselt number reaches a maximum at the inlet to the channel, then decreases, reaching a minimum at a distance of 2.0 mm from the inlet (i.e., at 2/3 of the channel length); in the final section, analogously to the previous case, it increases slightly due to the effect of the outlet vortex. At the point corresponding to the edge of the bow wave, a local minimum is visible on the entropy production line $S_{H}^{\prime }$, smaller than in Figure 3C. Again, it finds no counterpart in the Nusselt number distribution.

The comparison of the local entropy production due to friction and heat transfer (Figure 4D) shows that the entropy production due to heat transport again predominates; however, the entropy resulting from flow friction is already clearly visible, especially in the channel inlet region. The comparison of the entropy production distributions due to the flow friction in Figure 3B and Figure 4B shows an increase of two orders of magnitude due to the increase in the gas velocity from 0.3 m∙s^−1^ to 2 m∙s^−1^. At the same time, the entropy produced due to the heat transfer decreased almost twofold:$\;S_{H}≅$ 1∙10^−5^ W K^−1^ (cf. Figure 3D and Figure 4D). The cumulative entropy production (Figure 4E) shows a twofold decrease in the total entropy production in the reactor. Increasing the gas velocity in this case leads to a more efficient operating point, closer to the equilibrium state and causing less energy dissipation and thus leading to lower energy consumption in the reactor process.

Flow-temperature map, velocity $w_{0}=$ 6 m∙s^−1^ (Figure 5A). For this considerable velocity, inertial effects are significant. The outlet vortices are large, strongly deformed, and extend much further towards the channel inlet than in the previous cases. An additional, strongly deformed vortex is created outside the channel. The flow patterns that are formed show some similarity to the flow separation phenomenon, which usually occurs at much higher Reynolds numbers. The meeting point of the central fluid stream with the outlet vortex is shifted even further towards the channel inlet. Heat transport at the channel inlet is even more intensive. The hot spot is more spread out (on a longer section of the channel wall) and has a lower temperature (in all cases, the heat flux is 1500 W∙m^−2^ K^−1^, and the temperature scales in the figures differ).

Pressure and flow (Figure 5B). The flow velocity distribution is almost flat, the maximum is poorly visible. The static pressure reaches a minimum and stabilizes in the outlet part of the channel, in the area of the outlet vortex; there is a significant negative pressure here. The pressure gradient is the highest at the channel inlet, then decreases and stabilizes. At the channel inlet, a break is visible in the pressure, fluid velocity, and pressure gradient distribution lines, similar to Figure 3B and Figure 4B, corresponding to the edge of the bow wave. The distribution of entropy production due to the flow friction (Equation (4)) is analogous to Figure 4B, but its values are two orders of magnitude larger.

Heat transport (Figure 5C). The Nusselt number reaches a maximum at the channel inlet, then decreases, reaching a minimum at a distance of about 2.0 mm from the inlet; in the final section, analogously to the previous cases, it increases due to the effect of the outlet vortex. At the point corresponding to the edge of the bow wave, a local minimum is almost invisible on the entropy production line $S_{H}^{\prime }$.

The comparison of total entropy production due to friction and heat transfer (Figure 5E) shows that entropy production due to flow friction clearly predominates. As judging from the local entropy production (Figure 5D), entropy resulting from heat transfer is much smaller and occurs mainly in the outlet region of the channel, within the hot spot region, where the local Nusselt number shows a minimum. Entropy production due to flow friction takes place mainly within the inlet channel region (up to 1 mm of length), where the pressure gradient is the highest (cf. Figure 5B,D). Comparison of the entropy production distributions due to flow friction in Figure 4B and Figure 5B show an increase by two orders of magnitude. At the same time, entropy produced due to the heat transfer decreased relatively slightly, by about 30% (cf. Figure 4D and Figure 5D). Cumulative entropy production (Figure 5E) indicates a twofold increase in the total entropy production in the reactor compared to the gas velocity of 2 m∙s^−1^. Increasing the gas velocity in this case leads to reaching an unfavorable operating point, further from the equilibrium state, and causes greater energy dissipation and thus a greater energy consumption in the reactor process.

**Figure 6:**
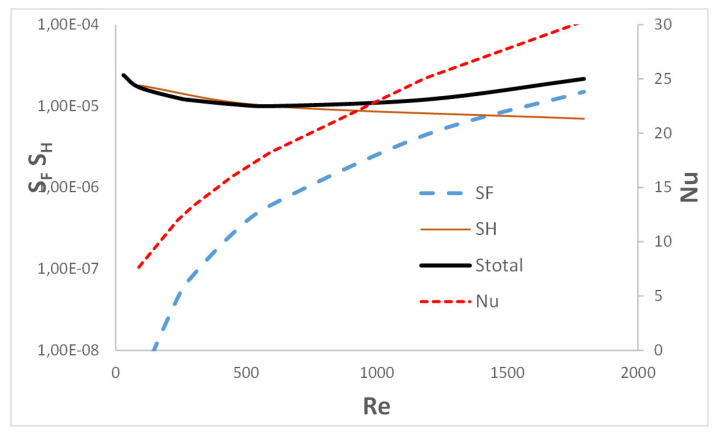
Entropy production vs. Reynolds number. $S_{F}$, $S_{H}$—entropy produced due to flow friction and mass transfer, respectively, W K^−1^. Total entropy $S_{total}=S_{F}+S_{H}$. Nusselt number refers to the right axis.

Entropy produced due to heat transfer, $S_{H}$, and due to flow friction, $S_{F}$, are plotted in Figure 6 vs. Reynolds number Re. The total entropy produced in the channel, i.e., the sum $S_{total}=S_{F}+S_{H}$, is also shown. The plot agrees well with the discussion presented above. For low velocity (or Reynolds number), a small Nusselt number causes a significant production of entropy due to heat transfer $S_{H}$. At higher speeds (i.e., higher Reynolds numbers), heat transport is more intensive and the Nusselt number is higher, which causes the entropy $S_{H}$ to decrease. At the same time, a higher velocity causes a very strong increase in flow resistance and, as a result, entropy, which is the result of flow friction, $S_{F}$. The total entropy production Stotal for small Reynolds numbers is close to the $S_{H}$ values, with increasing gas velocity it increases gradually (although relatively moderately), and for large flows it approaches the—dominant in this range—values of the entropy due to flow friction, $S_{F}$. Note that according to Equation (16), the $S_{H}$ entropy is inversely proportional to the heat transfer coefficient α (and thus to the Nusselt number).

## 6. Experimental Validation of the CFD Results

An important question arises: what is the accuracy of the research? Or even more important: what is the accuracy of the data derived from CFD simulations and substituted into the equations describing entropy production? To assess this accuracy, experimental results of heat transport in the structures discussed are presented.

In order to verify the results of CFD simulations of heat transport, a series of experiments were carried out. An air stream flowed through the tested streamlined structure under ambient conditions. The structure was heated by a high-intensity direct current (up to 350 A), flowing directly through the metal structure and heating it as a result of the Joule effect. The air stream temperatures at the inlet and outlet of the device were measured by thermocouples (a total of six thermocouples). The surface temperature of the structure was measured by thermocouples glued to the metal surface with an adhesive with excellent electrical insulation and good thermal conductivity. Four thermocouples were used on the inlet and outlet sides of the structure.

**Figure 7:**
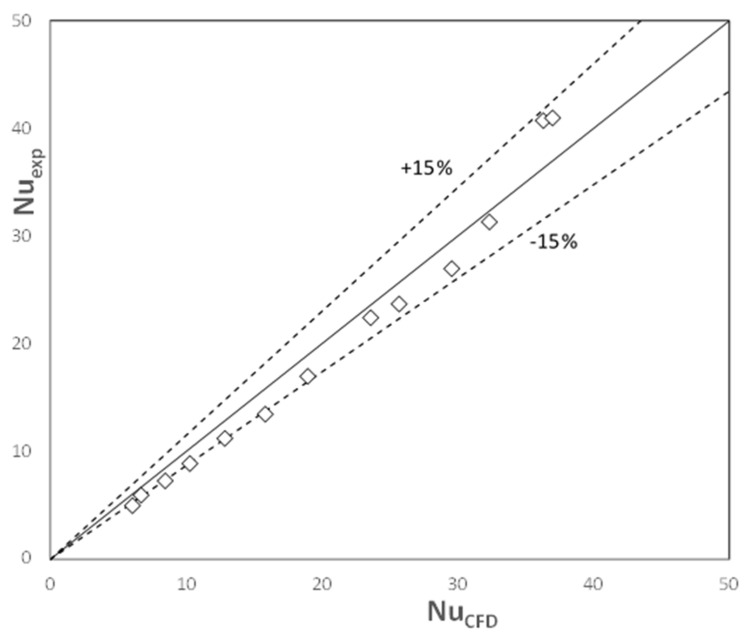
Experimental verification of the CFD-derived Nusselt numbers.

The results are shown in Figure 7. Satisfactory agreement between CFD calculations and experimental results was obtained (average relative error, ey = ±13%). It can therefore be assumed that the CFD procedure was carried out correctly.

## 7. Final Conclusions

The research results presented above allow the following general conclusions to be drawn.

1. The inlet vortex does not appear within the streamlined structure as a result of the rounded channel wall, leading to enhanced heat transfer compared to classic structures [8].
2. At the frontal, rounded surfaces of the channel, the intensive shearing flow causes intensive heat transport. In the inlet channel region, the entropy production by heat transport $S_{H}$ is low due to high Nusselt number. A large pressure gradient in this region produces large entropy due to friction $S_{F}$.
3. In the outlet channel region and beyond its end, outlet vortices are formed, leading to much less intensive heat (minimum of Nusselt number), a hot spot on the channel surface, and increased entropy production due to heat transfer $S_{H}$. The pressure gradient is close to zero here; thus, entropy due to friction almost disappears.
4. Flow resistance increases very strongly with the gas velocity (with the power of 2–3), causing a significant increase in the entropy production due to flow friction by orders of magnitude. The effect of gas velocity on the entropy due to heat transfer is opposite and much weaker (Nusselt increases with velocity with the power of 0.5–0.8). This trend is distinctly visible in Figure 6.
5. The total entropy production depends on the gas velocity (compare Figure 3, Figure 4 and Figure 5E). Both the lowest and highest gas velocities (0.3 m∙s^−1^ and 6 m∙s^−1^) cause similar entropy production, where the entropy from heat $S_{H}$. and flow $S_{F}$. dominates, respectively. For an intermediate velocity (2 m∙s^−1^), the entropy production is about twice as small. Proper selection of the operating conditions allows operation close to the equilibrium state, thus leading to low energy dissipation and high process efficiency. Based on Figure 6, the optimal operating point of the structure corresponds to Re≅ 600 (gas velocity $w_{0}≅$ 2 m s^−1^).

## Data Availability

Data can be accessed upon reasonable request.

## Abbreviations

A	channel cross-sectional surface area, m^2^
a	channel specific surface area, m^−1^
$c_{p}$	specific heat of the fluid, J∙kg^−1^ K^−1^
$D_{h}$	hydraulic diameter of the channel, m
F	inner surface area of the channel walls, m^2^
f	Fanning friction factor, dimensionless
$J_{n}$	stream (general)
$k_{n}$	proportionality factor (general)
L	channel length, m
$\dot{m}$	mass flow rate, kg s^−1^
Nu	Nusselt number, dimensionless
P	channel perimeter, m
p	pressure, Pa
q	heat flux, W m^2^
S	entropy, W K^−1^
T	temperature, K
$T_{b}$	fluid bulk temperature, K
$T_{in}$	inlet temperature, K
$T_{w}$	wall temperature, K
V	channel volume, m^3^
w	fluid velocity, m s^−1^
x	axial coordinate along the channel, m
y	transverse coordinate of the channel (perpendicular to the axis), m
	**Greek symbols**
α	heat transfer coefficient, W m^−2^ K^−1^
$\Delta \pi_{n}$	driving force (general)
ε	void fraction, dimensionless
λ	heat conduction coefficient, W m^−1^ K^−1^
ρ	fluid density, kg∙m^−3^
δ	boundary layer thickness, m
	**Subscripts**
F	refers to flow friction
H	refers to heat flow
i, j	refer to unit areas on the channel cross-section plane
